# Comprehensive Approach for the Genetic Diagnosis of Patients with Waardenburg Syndrome

**DOI:** 10.3390/jpm14090906

**Published:** 2024-08-27

**Authors:** Paula Inés Buonfiglio, Agustín Izquierdo, Mariela Vanina Pace, Sofia Grinberg, Vanesa Lotersztein, Paloma Brun, Carlos David Bruque, Ana Belén Elgoyhen, Viviana Dalamón

**Affiliations:** 1 Laboratory of Physiology and Genetics of Hearing, Institute of Genetic Engineering and Molecular Biology “Dr. Héctor N. Torres”—National Council of Scientific and Technology (INGEBI-CONICET), Buenos Aires C1428ADN, Argentina; paulabuonfiglio@gmail.com (P.I.B.); mpace@ingebi-conicet.gov.ar (M.V.P.); grinbergsofia@gmail.com (S.G.); abelgoyhen@gmail.com (A.B.E.); 2 Center for Endocrinological Research “Dr. César Bergadá” (CEDIE)—CONICET, FEI, Endocrinology División, Ricardo Gutiérrez Children’s Hospital, Buenos Aires C1425EFD, Argentina; aizquierdo@cedie.org.ar; 3 Translational Medicine Unit, Ricardo Gutiérrez Children’s Hospital, Buenos Aires C1425EFD, Argentina; 4 Patagonian Translational Knowledge Unit, El Calafate SAMIC High Complexity Hospital, El Calafate Z9405, Argentina; bruquecarlos@gmail.com; 5 Genetics Service, Central Military Hospital Surgeon General “Dr. Cosme Argerich”, Buenos Aires C1426, Argentina; vlotersztein@yahoo.com.ar; 6 “El Cruce” Néstor Carlos Kirchner High Complexity Hospital, Buenos Aires B1888, Argentina; brunpaloma@gmail.com; 7 Pharmacology Institute, Faculty of Medicine, University of Buenos Aires, Buenos Aires C1121A6B, Argentina

**Keywords:** Waardenburg syndrome, hearing loss, WES, CNVs, MLPA, genetic diagnosis

## Abstract

Waardenburg syndrome (WS) is a common genetic cause of syndromic hearing loss, accounting for 2–5% of congenital cases. It is characterized by hearing impairment and pigmentation abnormalities in the skin, hair, and eyes. Seven genes are associated with WS: *PAX*3, *MITF*, *EDNRB*, *EDN*3, *SOX*10, *KITLG*, and *SNAI*2. This study investigates the genetic causes of WS in three familial cases. Whole-exome sequencing (WES) was performed to identify single nucleotide variants (SNVs). Copy number variants (CNVs) were analyzed from the WES raw data and through multiplex ligation-dependent probe amplification (MLPA). The study identified one pathogenic SNV and two novel CNVs, corresponding to type I and type II WS patterns in the three families. The SNV, a nonsense variant (c.1198C>T p.Arg400*), was found in *MITF* and segregated in the affected father. The two CNVs were a deletion of exon 5 in *PAX*3 in a family with two affected members and a large novel deletion comprising seven genes, including *SOX*10, in a family with three affected members. These findings confirmed a WS diagnosis through genetic testing. The study emphasizes the importance of integrating multiple genetic testing approaches for accurate and reliable diagnosis, highlighting their role in improving patient management and providing tailored genetic counseling.

## 1. Introduction

Waardenburg syndrome (WS) (ORPHA:3440) is one of the most common syndromic forms of genetic hearing loss (HL), accounting for nearly 2–5% of congenital HL [1]. Given that the incomplete penetrance rate of the syndrome is nearly 20%, the current incidence is estimated to be 1/42,000 in the general population [2]. It is characterized by the presence of hearing impairment associated with pigmentation abnormalities, including depigmented patches of the skin and hair, vivid blue eyes, or heterochromia iridis. However, other features, such as dystopia canthorum, musculoskeletal abnormalities of the limbs, Hirschsprung disease (HD), or neurological defects, are found in subsets of patients and are used for the clinical classification of WS [2]. The association of hearing loss and the pigmentary abnormalities characteristic of WS results from an abnormal proliferation, survival, migration, or differentiation of neural crest-derived melanocytes [3]

WS is divided into four subtypes according to different concomitant phenotypes, and it is generally of autosomal dominant inheritance, but incomplete dominance or recessive cases can also be found in clinical practice [2,4]. The classification of WS is based on the presence of specific clinical features, in addition to pigmentary abnormalities and congenital sensorineural hearing loss, and type I and II are the most frequent subtypes. Type I WS (WS1) presents dystopia canthorum; type II WS (WS2) has no additional features; type III WS (WS3) includes both dystopia canthorum and musculoskeletal abnormalities of the upper limbs; and type IV WS (WS4) is associated with HD [5]. WS1 and 3 are characterized by dystopia canthorum (or telecanthus), a lateral displacement of the inner canthus of the eyes, which is considered to be the most reliable feature for WS1 classification due to its very high penetrance [6].

Hearing impairment is the most frequent feature and is generally bilateral (from 60% in WS1 to 90% in WS2) [4,5,7]. Its severity varies widely both within and between families, ranging from congenital, postlingual progressive hearing loss to profound deafness. Bilateral deafness is more frequent than unilateral and can be asymmetrical [6,8]. In WS2, the hearing defect is progressive in 70% of cases, and there is no typical audiogram shape [8]. A white forelock or premature graying of the hair before 30 years is present in at least one-third of both WS1 and 2 [2,9].

To date, seven genes have been associated with the different types of WS: endothelin receptor type B—*EDNRB* (WS4); KIT ligand—*KITLG* (WS2); melanocyte-inducing transcription factor—*MITF* (WS2); paired box 3—*PAX*3 (WS1, WS3); snail family transcriptional repressor 2—*SNAI*2 (WS2); SRY-box transcription factor 10—*SOX*10 (WS2, WS4); and endothelin 3—*EDN*3 (WS4). Disease-causing variants are mainly single nucleotide variants (SNVs), though copy number variants (CNVs) have also been reported [8,9]. Although de novo variants in sporadic cases of WS have been described in *MITF*, *PAX*3, and *SOX10*, a dominant pattern of inheritance is the most frequent [8,9].

The importance of describing both known and novel variants in genes previously associated with WS is often underestimated. Despite the lack of a definitive treatment for WS, in children with this disease, early diagnosis improves the clinical management of hearing impairment, which significantly impacts communication, speech, cognition, social interaction, and other aspects of the affected individual’s life [10,11]. In this regard, universal newborn screening enables the early detection of hearing impairment [12] before the appearance of other symptoms associated with WS. In particular, since 2001, Argentina has established a mandatory neonatal hearing screening program “https://www.argentina.gob.ar/normativa/nacional/66860/texto (accessed on 23 August 2024)”. Therefore, the early identification of causative variants in genes related to WS might anticipate other clinical manifestations, improve clinical management, and enable accurate genetic counseling [10]. Next-generation sequencing, especially whole-exome sequencing (WES), has accelerated the discovery of genes and variants that can elucidate the clinical diagnosis of WS, both in research settings and in clinical molecular diagnosis. WES has become an efficient and cost-effective alternative approach for the molecular diagnosis of this pathology since all the target genes can be studied at once. Genetic diagnosis allows differential diagnosis from similar pathologies, such as piebaldism, Tietz syndrome, and oculocutaneous albinism, among others. Therefore, accessing genetic tests and the consequent molecular diagnosis is essential and enriching for consistent genetic counseling for patients with WS and their families.

In this study, we identify the genetic etiology of WS in three familial cases with a dominant mode of inheritance, one presenting signs of type I WS and the other two of type II WS.

## 2. Methods

Three patients and their families, who were clinically suspected of having WS, were analyzed. The diagnoses were made by clinical geneticists based on the presentation of at least one major diagnostic criterion of the syndrome [1]: sensorineural hearing impairment, white forelock, pigmentary disturbance of the iris, or dystopia canthorum.

For all the samples, DNA extraction from peripheral blood lymphocytes was performed using the CTAB method [13]. DNA concentration and quality were evaluated using absorbance measurements at 260 nm and the ratios of absorbance at 260 nm/280 nm and 260 nm/230 nm, using a NanoDrop™ spectrophotometer (Thermo Fisher Scientific, Wilmington, NC, USA). The samples were stored at −20 °C. DNA integrity was further verified by 1% agarose gel electrophoresis using SYBR Safe DNA gel stain (Thermo Fisher Scientific, Wilmington, NC, USA). The gels were visualized using a transilluminator and image acquisition system (DNR Bio-Imaging Systems MiniBis Pro^®,^ Jerusalem, Israel).

**Figure 1:**
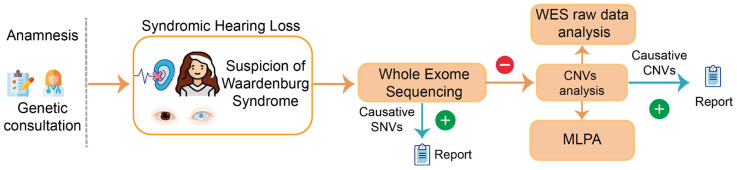
WS genetic diagnosis algorithm: after evaluation by a geneticist, causative SNVs were screened by WES. Patients with negative results were further studied for CNVs using bioinformatic algorithms from WES raw data and by MLPA. Some icons were obtained from Flaticon “https://www.flaticon.es (accessed on 23 August 2024)” and Biorender “https://www.biorender.com/ (accessed on 23 August 2024)” websites.

The proposed algorithm to achieve genetic diagnosis consisted of the following steps: (1) WES was performed as described in a previous report [14] for SNVs screening, filtering variants by the target genes, and the subsequent variant prioritization process; (2) when negative for SNVs, the CNVs were analyzed using the DECoN tool on the WES raw data; and (3) multiplex ligation-dependent probe amplification (MLPA) for P186-C3 *PAX*3, *MITF*, and *SOX*10-v01 was used to detect and/or confirm the presence of deletions in target genes in other family members (Figure 1).

### 2.1. WES Variant Prioritization Process

Variants from the seven genes reported to be causative of WS, *PAX*3, *MITF*, *EDNRB*, *ENDR*, *SOX*10, *KITLG*, and *SNAI*2, were selected by filtering with an in silico panel using a homemade Python script pipeline. This process included parameters such as mode of inheritance, variant localization, variant type (nonsynonymous variants, splice acceptor or donor site variants, and coding non-inframe in/dels), variant frequency in general population databases gnomAD “https://gnomad.broadinstitute.org/ (accessed on 23 August 2024)”, and published reports of pathogenicity and databases ClinVar https://www.ncbi.nlm.nih.gov/clinvar/ (accesed on 23 August 2024)”, among others. Pathogenicity prediction of the variants was performed with REVEL software “https://sites.google.com/site/revelgenomics (accesed on 23 August 2024)”. All the information was compiled, and the criteria rules were combined to reach a variant classification based on data retrieved from InterVar “http://wintervar.wglab.org/ (accesed on 23 August 2024)”, Varsome “https://varsome.com/ (accesed on 23 August 2024)”, and the Variant Interpretation Platform “http://hearing.genetics.bgi.com/ (accesed on 23 August 2024)”. Pathogenicity prediction and variant classification were rigorously reviewed and classified according to the American College of Medical Genetics and Genomics and the Association for Molecular Pathology (ACMG/AMP) guidelines and were further modified by taking into account the recommendations of the Hearing Loss Variant Curation Expert Panel (HL-VCEP) and the standards for CNVs interpretation using the ClinGen CNV Pathogenicity Calculator [15,16,17,18]. The variants classified as pathogenic and likely to be pathogenic were selected for segregation analysis within the family.

### 2.2. Copy Number Variants Analysis

#### 2.2.1. WES RAW Analysis

Different lengths of CNVs can be detected using various techniques. WES raw data analysis allows the detection of larger CNVs (even those extending beyond the gene under study). In contrast, MLPA enables the detection of smaller CNVs as a single exon to an entire gene, with high sensitivity. CNV detection from the WES raw data was performed using the DECoN (Detection of Exon Copy Number) software v1.0.1 tool [19]. Aligned sequencing reads in the BAM format were processed by selecting highly correlated reference samples, calculating the read depth for each exon, and normalizing against the reference set to account for sequencing biases. Bayes factors were calculated using DECoN to represent the likelihood ratio and to compare the probability of a CNV to the probability of a common copy number variation. This factor aids in annotating CNVs by providing a measure of evidence supporting the classification of these variants. The Bayes factor and reads ratio reported with the CNV list serve as indicators of confidence in a CNV call [19].

#### 2.2.2. MLPA Assays

To detect exon-level CNVs (deletions and duplications) with high sensitivity, MLPA was carried out. The protocol was performed according to the manufacturer’s manual (MRC-Holland, Amsterdam, the Netherlands) using Probemix P186-C3 PAX3 MITF SOX10-v01 to detect deletions or duplications in the *PAX*3, *MITF*, and *SOX*10 genes. The products were analyzed using a fragment analyzer sequencer (ABI 3730XL; Applied Biosystems, Foster City, CA, USA) with 500Liz as an internal size standard for fragment size determination. Data analysis was performed using the Coffalyser (MRC-Holland, Amsterdam, the Netherlands). Wild-type controls were included in all the reactions.

## 3. Results

All of the three families analyzed were diagnosed with heterozygous pathogenic variants in the studied WS target genes that were consistent with type I and type II WS. One of the variants was an SNV (the missense variant c.1198C>T p.Arg400* detected in *MITF* which segregated in the affected father). The other two families had novel CNVs: a small deletion of exon 5 in *PAX*3 in a family with two affected members and a large deletion encompassing the loss of several genes, including *SOX*10, which segregated in the three affected relatives of the family (Table 1).

### 3.1. Case #1

The proband, a 1-year-old boy, and his father had prelingual bilateral profound hearing loss. They had cochlear implants with good outcomes. Additionally, they both presented a white forelock of hair, which is consistent with type II WS.

**Figure 2:**
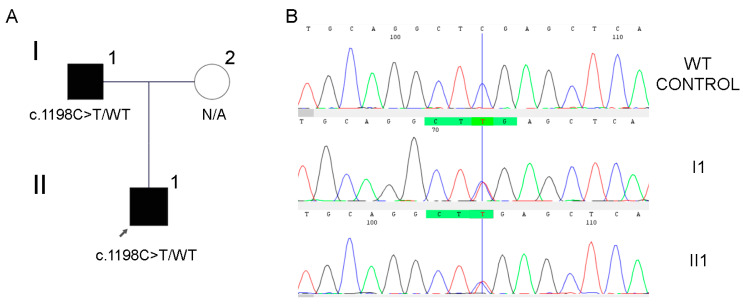
Pedigree and Sanger sequencing of the variant in *MITF* gene for family case #1. (**A**) Intrafamilial segregation of the variant (N/A: not available for the study). Squares are males and circle is female. The arrow indicates the proband patient. (**B**) Electropherogram by Sanger sequencing showing the heterozygous c.1198C>T variant in the affected members of the family and control wild type.

WES analysis was performed in the child, retrieving a total of 105,241 variants. After filtering by the 7 target genes for WS, 10 variants remained as candidates for further analysis. Considering the available information from databases and publications, a previously reported [21] variant in the *MITF* gene was prioritized: a heterozygous nonsense variant in exon 10 NM_001354604.2: c.1198C>T p.(Arg400*). The genetic variant was confirmed by Sanger sequencing in the proband, and segregation analysis was performed within the family, which showed that the candidate variant was also carried by his affected father, confirming the co-segregation of the variant with the pathology in the family (Figure 2). Applying the specific criteria of the ACMG and the HL-VCEP: PM2_Supporting (variant absent in gnomaD population database), PVS1_Strong (prediction of more than 10% of the protein loss), PS4_Supporting (three affected non-related probands carrying this variant), PP1_Strong (positive segregation), and PP4 (genotype–phenotype correlation), the variant was classified as pathogenic.

### 3.2. Case #2

A 12-year-old male with congenital profound bilateral hearing loss and his mother exhibited clinical signs consistent with type I WS. In addition, the boy had an intellectual developmental disorder and unprovoked aggressive outburst episodes. No causative or candidate SNVs in target genes were detected for WS by WES. In order to study the CNVs, both WES raw data analysis and MLPA analysis were performed.

**Figure 3:**
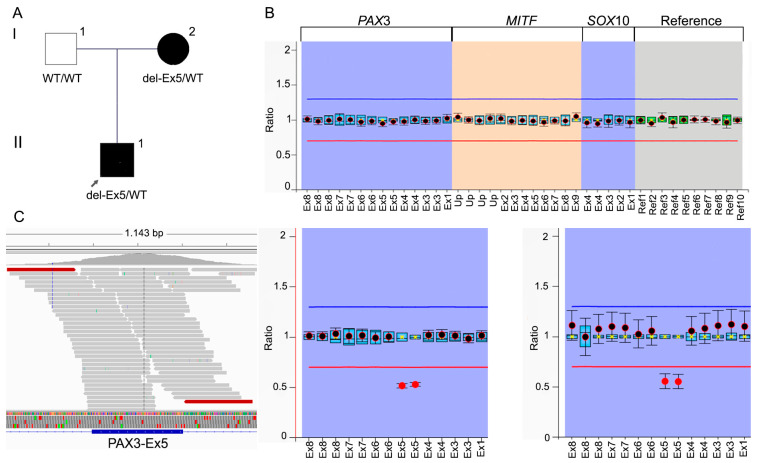
(**A**) Intrafamilial segregation of genetic alteration in *PAX*3. (**B**) Results obtained from the MLPA for the different probes to WS (Up: upstream). Top: control result for all the probes in the unaffected father. Bottom: zoom into the *PAX3* probes for the two affected relatives, showing a 0.5 ratio for the two probes of exon 5, indicating the heterozygous status of the deletion. (**C**) BAM file visualization of the proband demonstrating the absence of genetic variants in exon 5.

No CNVs were detected by the DECoN algorithm in the candidate genes in the analysis of the WES raw data. By MLPA analysis, a heterozygous novel deletion of *PAX*3 exon 5 was detected. MLPA segregation analysis confirmed the deletion in the affected mother as well; this was shown as a 0.5 ratio of the probes in exon 5 (Figure 3A,B). In order to rule out whether the exon appeared to be deleted due to the absence of the hybridization of any probe, which could be caused by a point variant in the region, the BAM file of sample II-1 was analyzed by looking for point variants in exon 5. Visualization of the BAM file from sample II-1 demonstrated the absence of any variant in exon 5, confirming the detected deletion of the entire exon (Figure 3C). Considering the probe-based description of the deletion, identified by MLPA, the CNV was NM_181458.4: c.(586+270_587-1)_(792+1_793-229)del p.(Ala196GlyfsTer4). Applying the ClinGen CNV Pathogenicity Calculator, the genetic alteration was classified as pathogenic (total score: 1.3) based on the following criteria: 1A. Contains protein-coding or other known functionally important elements; 2A. complete overlap of an established haploinsufficiency gene/genomic region, case–control and population evidence (ClinVar entries with at least 50%-80% reciprocal overlap variants reported as P/LP: ClinVar ID: 667019), and segregation with affected relatives.

### 3.3. Case #3

**Figure 4:**
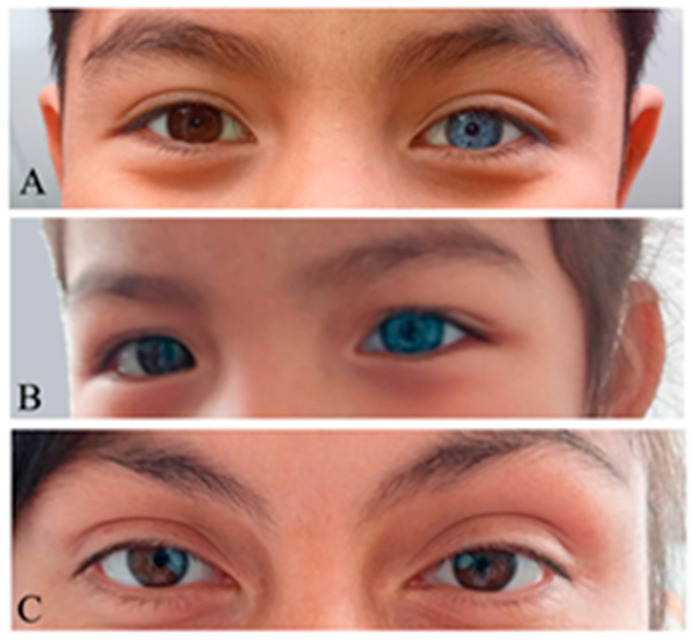
Full heterochromia in the siblings: (**A**) Proband (II-1). (**B**) Sibling (II-3). (**C**) Mother with segmental pigmentation of the iris (I-2).

This case involved two affected siblings and their affected mother with signs compatible with type II WS. The proband was a 7-year-old male (II-1) with prelingual severe to profound hearing loss and complete heterochromia (Figure 4A). His younger sister was 2 years old and exhibited prelingual severe hearing loss, including semicircular canal malformation and complete heterochromia (II-3) (Figure 4B). Their mother exhibited unilateral hearing loss and partial heterochromia (Figure 4C). All were equipped with hearing aids and both affected siblings will undergo cochlear implantation.

**Figure 5:**
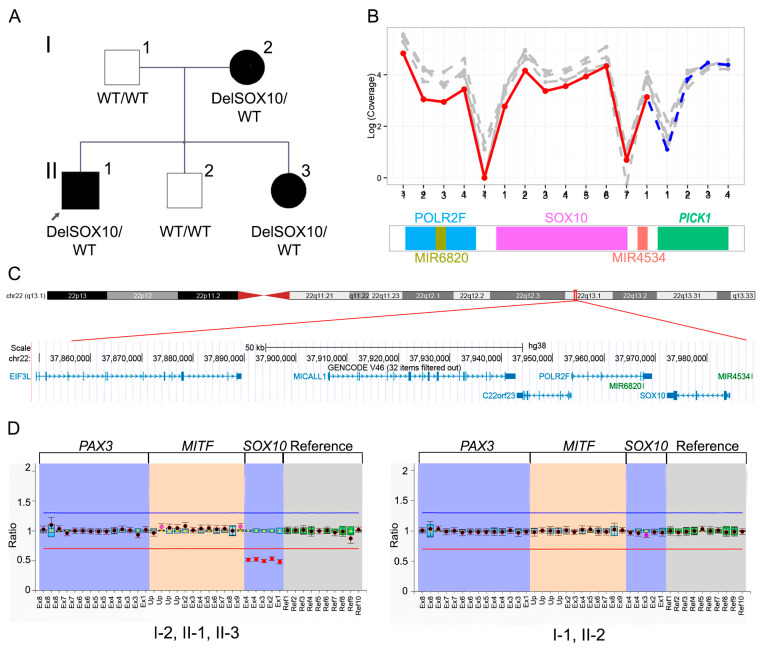
(**A**) Intrafamilial segregation of *SOX*10 deletion in family #3 with WS. (**B**) Top: the plot shows the log-normalized (coverage) of the sample of interest (blue) relative to reference samples (gray). Deleted exons are in red. The plot represents the log (coverage) vs. the number of probes for each gene analyzed. Below: scheme showing a subregion of the deletion with the genes flanking *SOX*10. (**C**) Visualization in the UCSC Genome Browser on Human (GRCh38/hg38) of the large deletion detected by WES raw data analysis. (**D**) MLPA results for all the family members demonstrating the heterozygous deletion of the entire *SOX*10 gene in the affected relatives.

No SNVs were detected in the proband by WES. As a result of the WES raw data analysis, a large novel heterozygous deletion with a Bayes factor of 158 was identified in the male proband and involved seven genes: *EIF*3*L*, *MICALL*1, *C*22*orf*23, *POLR*2*F*, *MIR*6820, *MIR*4534, and *SOX*10. Considering the DECoN results, the deletion was seq[GRCh38] del(22)(22p13.1)NC_000022.11:g.(?_37849420)_(37988853_?)del. The genomic position of the deletion is Chr22:37849420-37988853 and includes at least 139 Kb of the length. The BAM file was analyzed without restriction of the capture targets in order to identify the breakpoints, with inconclusive results. An analysis of the short tandem repeats in the area remains to be studied, to identify the breakpoints. Notably, except for the *SOX10* gene (related to type II WS), the remaining lost genes have not been related to any human disease. This is in accordance with the observation that the affected family members only exhibited signs compatible with WS. The complete loss of *SOX10* was confirmed by MLPA and was also segregated in the affected family members (Figure 5). Applying the ClinGen CNV Pathogenicity Calculator, the genetic alteration was classified as pathogenic (total score: 1.45) based on the following criteria: 1A. Contains protein-coding or other known functionally important elements; 2A. complete overlap of an established haploinsufficiency gene/genomic region, case–control and population evidence (ClinVar entries with at least 50–80% reciprocal overlap deletions reported as P/LP: ClinVar ID: 545010; 57629; 153362; 57632; 156718), and segregation with affected relatives.

## 4. Discussion

Waardenburg syndrome is the most common syndromic genetic disease associated with congenital hearing loss. In this study, we identified the genetic etiology of three families with different types of WS. This work represents a pivotal contribution to the broadening of the spectrum of Waardenburg syndrome genetics in Argentina. By providing a comprehensive analysis of genotype–phenotype correlations using various molecular biology techniques, our work offers valuable insights into the local genetic landscape of Waardenburg syndrome. To our knowledge, only one previous study involving an Argentine proband with WS type 2A was reported, though it was conducted abroad [22]. Our research thus lays an important foundation for further exploration of this condition within Argentina. In the first case, a nonsense variant in the *MITF* gene was detected. This variant was located in the last exon of *MITF*; therefore, the PVS1 criteria was adjusted to Strong since it was predicted to lose more than 10% of the protein. In this regard, based on the published data, the mechanism for the disease is likely to be haploinsufficiency [23,24,25]. The identified variant was first reported in a family with WS [21]. In that case, the five affected relatives exhibited a range of features, such as normal hearing to severe hearing loss and full and partial heterochromia iridis, and all presented pigmentary disturbances of the hair, such as a white forelock. In comparison, the proband of case #1 had congenital profound hearing loss and a white forelock, but no iridis pigmentary disturbances. An additional family carrying this variant had severe to profound hearing loss with a dominant mode of inheritance, without other clinical signs [14]. This finding is in accordance with some reports where *MITF* gene variants cause only hearing loss [26,27]. The variable phenotype expression of the c.1198C>T variant in *MITF*, from non-syndromic to syndromic cases, could be explained by the presence of modifier genes, as well as interactions with environmental factors [28,29]. Likewise, a polygenic background has been proposed to explain inter- and intra-familial variability expression, as a pair of monozygotic twins exhibited the exact same WS phenotype, with remarkably similar audiograms, while the other affected relatives presented differences in the phenotype expression [30]. Nevertheless, stochastic molecular events are also suggested as a contributing factor to variable expressivity, such as NMD efficiency, age, sex, cis/trans elements, and epigenetic modifications [31]. For instance, DNA methylation plays a critical role in regulating tissue-specific gene expression, alternative splicing, the prevention of transcription from cryptic promoters, and X chromosome inactivation, all of which influence disease progression [32]. In this regard, genotype–phenotype association analyses are needed in order to identify the correlations between the specific genes associated with WS and its various clinical manifestations [33,34].

In the other two diagnosed cases, a novel deletion of a single exon in *PAX*3 or an entire *SOX*10 gene loss were identified. Several reports showcased the presence of CNVs as a genetic cause of different types of WS. For the *PAX*3 gene, CNVs have been identified as causative variants in 6% of diagnosed probands [6]. Thus, the search for CNVs increases the diagnostic yield of WS [35]. Haploinsufficiency in these two genes has been previously described as a disease mechanism [8,36,37,38]. Likewise, when considering genetic constraints, a measure of how much a genomic region is under negative selection, both genes show high intolerance to loss-of-function variants. Specifically, *PAX*3 has a probability of loss-of-function intolerance (pLI) of 1 and an observed/expected ratio (o/e) of 0.26 (0.17–0.41), while *SOX*10 also has a pLI of 1 and an o/e of 0.1 (0.04–0.25) [39,40]. These findings are consistent with the results obtained for cases 2 and 3.

In case #2, a deletion of exon 5 was identified in *PAX*3 in both the proband and his affected mother; this is in accordance with the type 1 WS features exhibited in the affected relatives. This deletion was detected by MLPA, but it was unobserved throughout the DECoN algorithm. Although DECoN is generally highly sensitive and specific, the detection of small CNVs, particularly single-exon deletions, can still be challenging due to limited read depth and variability in coverage across exons [41,42]. A study evaluating the performance of CNV analysis from NGS gene panels indicates that while the algorithm generally performed well, single-exon deletions are sometimes missed, particularly in regions with low coverage or poor probe design [43]. These limitations underscore the importance of validating DECoN findings with additional methods such as MLPA to ensure accuracy and avoid false negatives. The intellectual disability described in the proband is in accordance with some reports of type 1 and type 3 WS, accompanied by mental retardation, autistic spectrum disorder (ASD), and behavioral problems [44,45,46].

In case #3, with type II WS, a large deletion was identified in the proband involving the loss of seven genes, including the entire *SOX*10 gene. The fact that the other six genes have not been linked to any human diseases is consistent with the observation that the affected families exhibited only signs related to WS. This exact deletion has never been described before and is not present in the ClinVar database. However, five pathogenic deletions larger than, but including, the novel deletion detected in case #3 are reported in ClinVar (IDs: 545010, 57629, 153362, 57632, 156718). These deletions have been associated with Waardenburg syndrome type 4, Hirschsprung disease, hearing impairment, and global developmental delay in ClinVar. Accompanying publications that conduct a deep genotype–phenotype correlation of these five deletions are not available. However, since the overlapping region of all five ClinVar reports and that of case #3 of the present work include the loss of the *SOX*10 gene, it is the loss of this gene that is the most likely underlying cause of WS in these patients. Thus, we provide new and significant evidence of a novel large deletion associated with WS, and our findings can be extended to those reported in ClinVar. Notably, the affected sister (II-3) had semicircular canal malformation, which is particularly associated with genetic variants in *SOX*10 [8]. Thus, some studies have demonstrated a strong association between *SOX*10 variants and various inner ear malformations in WS patients, such as hypoplasia or agenesis of the semicircular canals, an enlarged vestibular aqueduct, and cochlear deformities [47,48,49]. It should be noted that although unilateral HL is more commonly associated with type I WS [8,34], the mother (I-2) exhibited unilateral hearing loss, thus indicating variability in the phenotypic expression of the syndrome.

Early identification and accurate diagnosis enable interventions that will not only improve hearing functions, but also the general quality of life. Therapeutic options for cases with profound hearing loss typically focus on amplification devices or cochlear implants, which serve as a definitive and effective surgical treatment. Cochlear implantation in congenitally deaf children with WS is a well-established intervention as a method of auditory rehabilitation, demonstrating improved audiometry, speech perception, and speech intelligibility [50]. Patients with WS typically achieve favorable outcomes following cochlear implant surgery, even in patients with temporal abnormalities [7,50]. Likewise, the proband of case #1 had cochlear implants with excellent outcomes. Moreover, both affected siblings of case #3 will undergo surgery for cochlear implants. Based on the confirmed genetic diagnosis of WS, a favorable outcome of this intervention is expected.

The present results highlight the importance of combining different molecular biology and bioinformatics strategies to achieve an accurate diagnosis of WS leading to accurate genetic counseling.

## Figures and Tables

**Table 1:** Clinical features and genetic classification of patients: Each row describes a patient case, specifying the affected gene, the WS subtype, the genotype according to the RefSeq reference, and the observed clinical features. The pathogenicity classification is based on the criteria established by the ACMG and HL-VCEP, indicating the points and categories of evidence supporting the classification. The genes in bold are OMIM genes, and the notation del-Ex5 is an abbreviated form, where the symbol # represents the notation c.(586+270_587-1)_(792+1_793-229)del [20].

Case	Gene	WS Subtype	Genotype (RefSeq NM)	Proband’s Clinical Features	Classification According to ACMG and HL-VCEP
1	***MITF***	Type II	c.1198C>T (NM_001354604.2) p.(Arg400*)	White forelock Congenital profound sensorineural hearing loss Cochlear implants	Pathogenic (PM2_Supporting, PVS1_Strong, PS4_Supporting, PP1_Strong, PP4) 11 points = 11 P-0B
2	***PAX*3**	Type I	del-Ex5 ^#^ (NM_181458.4)	Dystopia canthorum Hypoplastic blue eyes Congenital profound sensorineural hearing loss Developmental disorder	Pathogenic Total score: 1.3
3	*EIF*3*L*, *MICALL*1, *C*22*orf*23, *POLR*2*F*, *MIR*6820, *MIR*4534, ***SOX*10**	Type II	seq[GRCh38] del(22)(22p13.1) NC_000022.11:g.(?_37849420)_(37988853_?)del	Heterochromia iridis Congenital profound sensorineural hearing loss	Pathogenic Total score: 1.45

## Data Availability

The original contributions presented in the study are included in the article.
